# The role of PPARγ in cancer cachexia: friend or foe?

**DOI:** 10.3389/fendo.2025.1717134

**Published:** 2025-12-16

**Authors:** Leili Ding, Hao Jiang, Liang Shen, Yiming Xu

**Affiliations:** 1 Department of Thoracic Surgery, Southeast University Affiliated Nantong First People’s Hospital, Affiliated Hospital 2 of Nantong University, Nantong First People’s Hospital, Nantong, China; 2 School of Medicine, Nantong University, Nantong, China; 3 Department of General Surgery, Haimen Traditional Chinese Medicine Hospital, Haimen, Jiangsu, China

**Keywords:** PPARγ, cancer cachexia, lipolysis, muscle wasting, inflammation, TZDs

## Abstract

Cachexia remains a major complication in cancer, with limited therapeutic options. Peroxisome proliferator-activated receptor gamma (PPARγ) has emerged as a key regulator of adipogenesis, lipid metabolism, and inflammation, but its role in cachexia is paradoxical. PPARγ activation can promote lipid storage, suppress inflammation, and modulate muscle–adipose crosstalk, potentially alleviating tissue wasting. Conversely, PPARγ agonists may enhance tumor growth in certain cancers, raising safety concerns. This review examines the dual functions of PPARγ in cancer cachexia, focusing on its regulation of adipose tissue remodeling (including browning and lipid metabolism), skeletal muscle homeostasis, and systemic inflammation, alongside tumor-promoting mechanisms that complicate its therapeutic use. Finally, emerging approaches such as selective PPARγ modulators (SPPARγMs) and tissue-targeted strategies are discussed to maximize anti-cachectic effects while minimizing oncogenic risks. Understanding these context-dependent actions is essential for translating PPARγ modulation into safe, effective cachexia therapies.

## Introduction

Cancer cachexia is a multifactorial syndrome characterized by severe weight loss, skeletal muscle atrophy, and adipose tissue remodeling, accompanied by systemic inflammation and profound metabolic dysregulation (1). Unlike simple starvation, cachexia is refractory to nutritional support and contributes to increased morbidity, diminished therapeutic efficacy, and poor survival in patients with advanced malignancies. It affects up to 80% of cancer patients, particularly those with pancreatic, gastric, lung, and colorectal cancers, and accounts for approximately 20% of cancer-related mortality (2, 3). Clinically, cancer cachexia progresses through three stages: precachexia, cachexia, and refractory cachexia. Precachexia is marked by early metabolic alterations, such as anorexia and impaired glucose tolerance, with a body weight loss of 5% or less. Cachexia is diagnosed in patients who experience weight loss exceeding 5%, or more than 2% in individuals already underweight (body mass index [BMI] < 20 kg/m²) or with low skeletal muscle mass. The refractory stage is characterized by progressive, treatment-resistant wasting in the context of advanced, unresponsive malignancy (4). The pathogenesis of cancer cachexia involves a complex interplay between tumor-derived factors and host responses, resulting in hypercatabolism of both muscle and fat, hormonal dysregulation, and persistent systemic inflammation (3, 5, 6). In light of the limited therapeutic options currently available, there is an urgent need to identify novel molecular targets to counteract the progressive tissue wasting and metabolic disturbances characteristic of this syndrome.

PPARγ is a nuclear receptor and transcription factor that plays a central role in regulating lipid metabolism, adipocyte differentiation, insulin sensitivity, and inflammation (7, 8). Upon activation by endogenous ligands or synthetic agonists, PPARγ modulates transcriptional networks that promote energy storage, enhance insulin sensitivity, and exert anti-inflammatory effects (9, 10). Given its diverse functions, PPARγ serves as a critical regulator of metabolic homeostasis and may influence both the development and potential therapeutic modulation of cancer cachexia.

Emerging evidence highlights the paradoxical role of PPARγ in cancer cachexia. On one hand, PPARγ activation may attenuate cachexia-associated wasting by preserving adipose tissue function, suppressing systemic inflammation, and protecting skeletal muscle integrity (11–13). On the other hand, concerns persist regarding its potential tumor-promoting effects in certain malignancies, as well as its context-dependent influence on energy expenditure, adipose tissue browning, and lipolysis. For example, PPARγ has been implicated in the progression of metastatic prostate cancer through the activation of lipid signaling pathways, including the upregulation of lipid synthesis enzymes (14). Additionally, PPARγ activation may exert pro-tumorigenic effects within the tumor microenvironment, particularly through modulation of myeloid cells. However, recent clinical studies have also demonstrated potential therapeutic benefits of PPARγ agonists in lung cancer, further illustrating its complex, tissue-specific actions (15). This dualistic nature, protective in peripheral metabolic tissues yet potentially detrimental within the tumor microenvironment, underscores the challenges of therapeutically targeting PPARγ in cancer. In this review, we examine the emerging evidence on PPARγ’s dual role in cancer cachexia, focusing on its mechanistic contributions to adipose and muscle remodeling, its interactions with inflammatory and metabolic pathways, and the therapeutic opportunities and risks associated with modulating its activity. A more nuanced understanding of the context-dependent functions of PPARγ may reveal novel avenues for selectively harnessing its benefits while minimizing its oncogenic potential in the management of cancer cachexia.

## Overview of PPARγ: structure, activation, and function

To contextualize the role of PPARγ in cancer cachexia, it is essential to first examine its structural features, mechanisms of activation, and physiological functions. PPARγ, a member of the nuclear receptor superfamily of ligand-activated transcription factors, plays a pivotal role in regulating energy homeostasis, lipid metabolism, and inflammatory responses. Owing to its multifaceted regulatory functions, PPARγ has garnered significant interest in cancer cachexia.

### Molecular structure and isoforms

**Figure 1:**
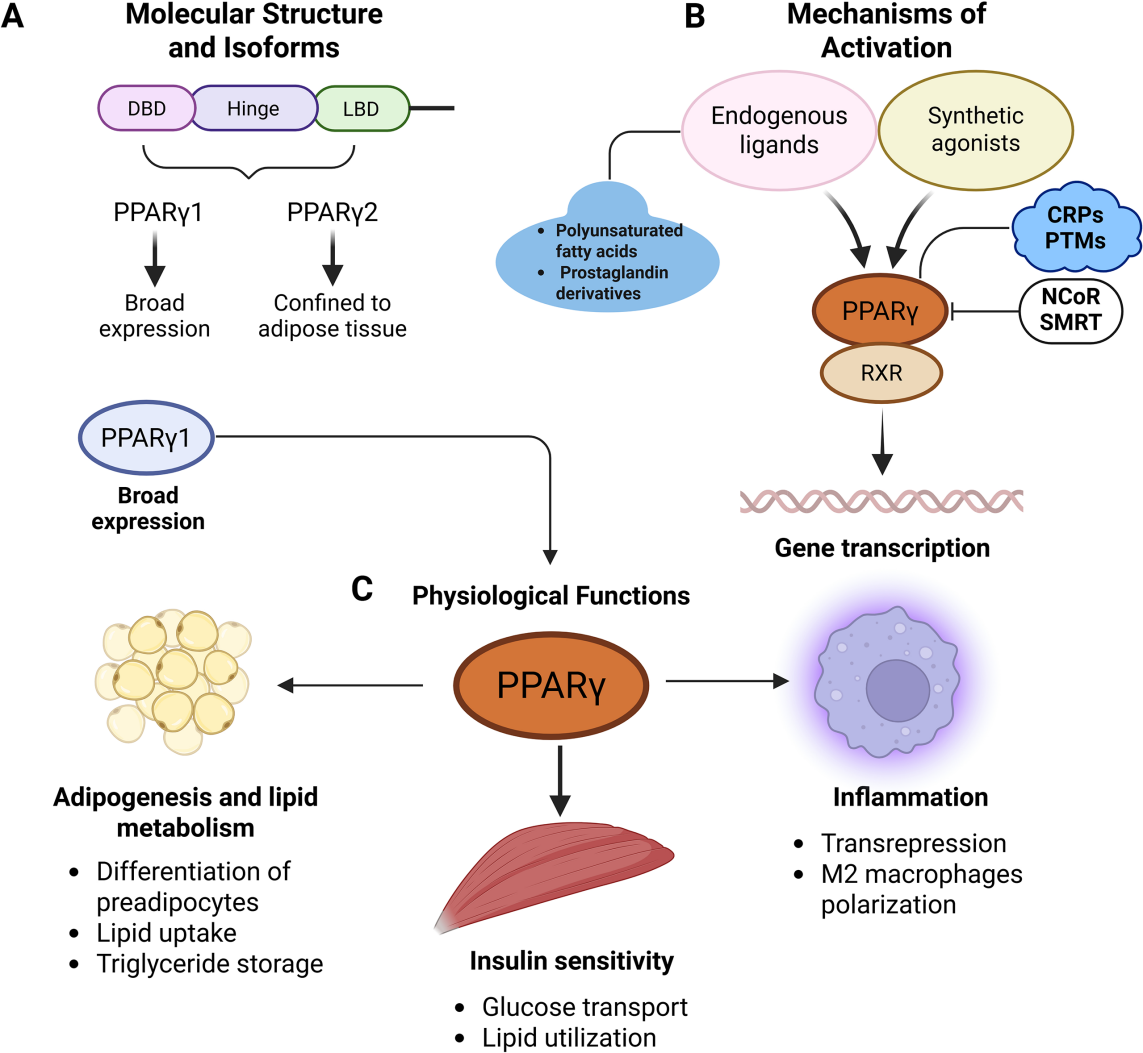
Structure, activation, and functions of PPARγ. **(A)** PPARγ, which exists as two isoforms, PPARγ1 (broadly expressed) and PPARγ2 (adipose-specific), is a nuclear receptor composed of a DBD, hinge region, and ligand-binding domain. **(B)** Upon activation by endogenous or synthetic ligands, PPARγ forms a heterodimer with RXR and binds to PPREs, where its transcriptional activity is fine-tuned by co-regulators and PTMs. **(C)** Through this regulatory network, PPARγ governs adipogenesis and lipid metabolism, improves insulin sensitivity, and exerts potent anti-inflammatory effects.

PPARγ exists in two principal isoforms, PPARγ1 and PPARγ2, which arise from alternative promoter usage and differential splicing of the PPARG gene (16, 17). These isoforms share a conserved modular structure typical of nuclear receptors (NRs), comprising a DNA-binding domain (DBD) with two zinc finger motifs, a flexible hinge region, and a LBD that facilitates ligand-dependent transcriptional regulation (18). Notably, PPARγ2 possesses an additional 30-amino-acid N-terminal extension, which confers enhanced transcriptional activity, particularly in adipose tissue (17, 19). In terms of tissue distribution, PPARγ1 is broadly expressed across various organs, including the liver, heart, and skeletal muscle, reflecting its systemic metabolic functions. In contrast, PPARγ2 expression is largely confined to adipose tissue, where it plays a central role in adipogenesis and lipid handling (20, 21). This tissue-specific expression pattern underpins the diverse yet interrelated biological functions of the two isoforms, As shown in **Figure 1A**.

### Mechanisms of activation

PPARγ functions as a ligand-activated transcription factor. Upon ligand binding, PPARγ forms a heterodimer with retinoid X receptor (RXR) and binds to peroxisome proliferator response elements (PPREs) in the promoter regions of target genes, thereby modulating gene transcription (22, 23). Activation of PPARγ is regulated by a range of endogenous ligands, including polyunsaturated fatty acids, prostaglandin derivatives such as 15-deoxy-Δ12,14-prostaglandin J2 (15d-PGJ2), and oxidized phospholipids (24, 25). In addition, synthetic agonists, particularly thiazolidinediones (TZDs) like rosiglitazone and pioglitazone, have been widely used as insulin sensitizers in type 2 diabetes and as pharmacological tools in preclinical cachexia studies (26, 27). PPARγ activity is also tightly regulated by co-regulatory proteins (CRPs) and post-translational modifications (PTMs). Coactivators such as Peroxisome proliferator-activated receptor gamma coactivator-1 alpha (PGC-1α) enhance its transcriptional activity, while corepressors like NCoR (nuclear receptor corepressor) and SMRT (silencing mediator of retinoic acid and thyroid hormone receptor) repress it (28, 29). PTMs, including phosphorylation, SUMOylation, and ubiquitination, modulate the stability, localization, and transcriptional efficacy of PPARγ (30, 31). These modifications provide context-specific control of its metabolic and anti-inflammatory actions under both physiological and pathological conditions (**Figure 1B**).

### Physiological functions

PPARγ is a master regulator of adipogenesis and lipid metabolism. It governs the differentiation of preadipocytes into mature adipocytes by upregulating key adipogenic genes, including fatty acid binding protein 4 (FABP4), adiponectin (APN), and lipoprotein lipase (LPL) (23, 32). In mature adipocytes, it facilitates lipid uptake and triglyceride storage, thereby maintaining lipid homeostasis and preventing ectopic lipid deposition in non-adipose tissues (21). Furthermore, PPARγ exerts systemic metabolic effects by enhancing insulin sensitivity through transcriptional regulation of genes involved in glucose transport and lipid utilization, such as Glucose Transporter Type 4 (GLUT4), Insulin Receptor Substrate 1 (IRS1), and Cluster of Differentiation 36 (CD36) (30). These metabolic actions are complemented by its potent anti-inflammatory properties. Specifically, PPARγ can inhibit the activity of key pro-inflammatory transcription factors, including nuclear factor kappa B (NF-κB), Signal Transducer and Activator of Transcription (STAT), and Activator Protein 1 (AP-1), through mechanisms of transrepression (33, 34). Additionally, it modulates immune cell phenotypes, particularly promoting the anti-inflammatory M2 polarization of macrophages (35). Emerging evidence also implicates PPARγ in skeletal muscle biology, where it contributes to muscle fiber type switching and suppresses myosteatosis, both of which are relevant to muscle wasting conditions such as cachexia (36). Thus, PPARγ serves as a critical node at the intersection of metabolic regulation and inflammation (**Figure 1C**).

### Mechanisms of PPARγ in cancer cachexia

In cancer cachexia, PPARγ primarily functions as an anti-catabolic and anti-inflammatory regulator, safeguarding skeletal muscle and adipose tissue from excessive breakdown, suppressing systemic inflammation, and modulating tumor-host metabolic cross-talk and central nervous system (CNS) homeostasis.

### PPARγ and adipose tissue remodeling

A hallmark of cancer cachexia is excessive lipid mobilization driven by accelerated lipolysis, which leads to profound energy depletion and exacerbates systemic energy imbalance. This catabolic process is mediated largely by the upregulation of the lipolytic enzymes adipose triglyceride lipase (ATGL) and hormone-sensitive lipase (HSL), both of which hydrolyze triglycerides into free fatty acids (37–39). Concomitantly, suppression of PPARγ, a nuclear receptor essential for adipocyte differentiation and lipid storage, further promotes adipose tissue wasting (36, 39). Tumor-derived extracellular vesicles (EVs), enriched with bioactive molecules such as microRNAs, have also been shown to downregulate PPARγ, thereby amplifying adipose tissue catabolism (37, 40, 41).

Pharmacological activation of PPARγ exerts protective effects by repressing these lipolytic programs. Agonists such as rosiglitazone and pioglitazone reduce ATGL and HSL expression and activity, thereby limiting triglyceride hydrolysis and preserving lipid reserves (11, 42, 43). Through this mechanism, PPARγ helps to maintain systemic energy homeostasis and slow the progression of cachexia-induced fat loss. Beyond lipolysis, cachexia also drives phenotypic remodeling of white adipose tissue (WAT) toward a brown-like state, characterized by enhanced mitochondrial biogenesis and increased expression of thermogenic genes such as uncoupling protein 1 (UCP1) and PGC1α (44–47). This “browning” process, when driven by pro-inflammatory and tumor-derived factors, including IL-6, TNF-α, and PTHrP, is maladaptive, resulting in uncontrolled energy dissipation and metabolic inefficiency (47–49). In this pathological context, PPARγ expression and activity are frequently reduced, contributing to the loss of mature adipocyte identity and to energy-wasting phenotypes (48, 50, 51).

**Figure 2:**
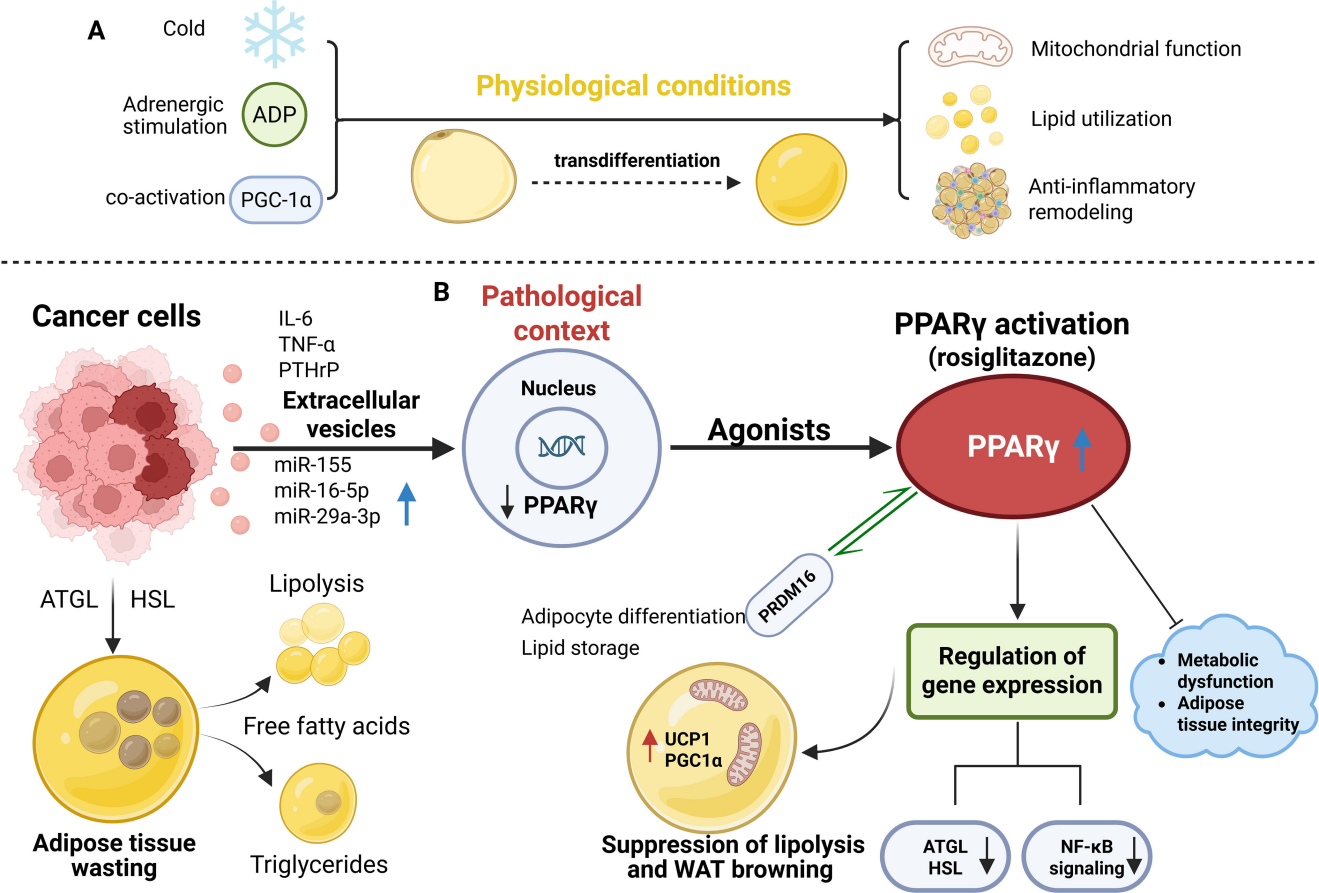
PPARγ in the regulation of adipose tissue homeostasis. **(A)**. Under physiological conditions, stimuli such as cold and adrenergic stimulation promote the transdifferentiation of adipocytes, leading to improved mitochondrial function, lipid utilization, and anti-inflammatory remodeling. **(B)**. In cancer cachexia, tumor-derived extracellular vesicles (EVs) and microRNAs (miRs) suppress PPARγ, driving excessive lipolysis and adipose wasting. Activation of PPARγ by pharmacological agonists such as rosiglitazone counteracts these effects by suppressing ATGL/HSL-mediated lipolysis, alleviating metabolic dysfunction and NF-κB-dependent inflammatory signaling, and promoting thermogenic gene programs, including the induction of PGC1α and UCP1.

Importantly, PPARγ does not uniformly inhibit adipose browning; rather, its actions are highly context-dependent (51, 52). Under physiological conditions such as cold exposure, adrenergic stimulation, or co-activation with PGC-1α, PPARγ promotes the transdifferentiation of white adipocytes into metabolically active beige adipocytes (51, 52). This adaptive browning is associated with improved mitochondrial function, enhanced lipid utilization, and anti-inflammatory remodeling of adipose tissue (53, 54). Conversely, in cancer cachexia, pathological browning arises in a pro-inflammatory, catabolic milieu where PPARγ signaling is suppressed (47–49, 55). In this setting, pharmacological activation of PPARγ may restore adipogenic differentiation and lipid storage capacity, mitigating inflammatory stress and maladaptive thermogenesis rather than simply “inhibiting browning.” Through interactions with co-regulators such as PRDM16, PPARγ orchestrates a delicate balance between adipogenesis, thermogenesis, and metabolic adaptation (56). Accordingly, activation of PPARγ in cachexia models has been shown to attenuate cytokine-driven WAT browning, preserve lipid stores, and restore a more regulated, metabolically competent adipose phenotype (11, 57, 58). Although clinical data remain limited, emerging studies suggest that PPARγ-targeted therapies may ameliorate metabolic dysfunction and maintain adipose tissue integrity in patients with cancer cachexia (57, 59). Taken together, PPARγ emerges as a multifaceted regulator of adipose tissue homeostasis in cancer cachexia, integrating lipid metabolism, inflammatory signaling, and thermogenic control. Its activation may not simply repress browning but rather reestablish a physiological equilibrium between energy storage and expenditure (**Figure 2**).

### PPARγ and skeletal muscle preservation

Although PPARγ is primarily expressed in adipocytes, its regulatory influence extends well beyond adipose tissue, exerting systemic effects that critically modulate skeletal muscle homeostasis (30, 60). Evidence from tumor-bearing mouse models demonstrates that administration of the PPARγ agonist GW1929 (10 mg/kg body weight) significantly attenuates muscle wasting during experimental cachexia (61). The protective effects of PPARγ activation are largely attributed to its metabolic, endocrine, and anti-inflammatory properties, which facilitate adipose-muscle crosstalk. In particular, PPARγ enhances APN secretion, which activates AMP-activated protein kinase (AMPK) and AKT signaling in skeletal muscle, thereby stimulating anabolic pathways, increasing protein synthesis, and improving mitochondrial energy metabolism (11, 30, 62, 63). These adaptations not only enhance energy availability but also reduce the catabolic stress that drives proteolysis in cachectic states. Mechanistically, PPARγ activation suppresses the expression of muscle-specific E3 ubiquitin ligases, Muscle RING-finger protein-1 (MuRF1) and Atrogin-1, both of which are central mediators of proteasome-dependent protein degradation (64). Moreover, PPARγ signaling inhibits NF-κB-dependent transcriptional activation in skeletal muscle, mitigating inflammation-associated muscle abnormalities (65). In non-cancer models of muscle atrophy, such as diabetes-induced sarcopenia (66) and angiotensin II-induced wasting (64, 67), PPARγ expression is suppressed, whereas its pharmacological activation alleviates muscle loss and promotes regeneration following injury (68). Collectively, the evidence identifies PPARγ as a central regulator of anti-catabolic signaling, crucial for preserving skeletal muscle integrity in cachexia.

**Figure 3:**
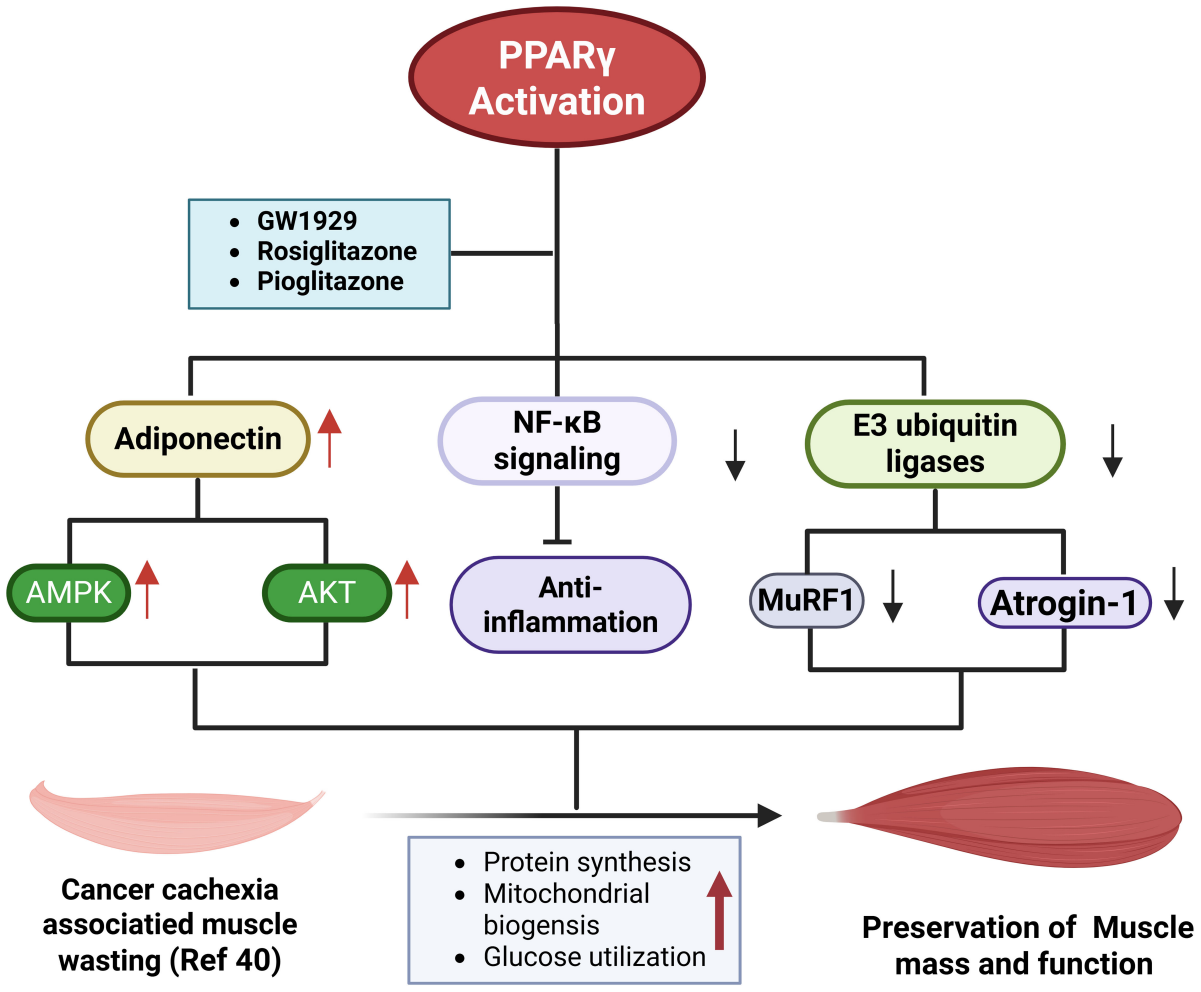
PPARγ activation in mitigating muscle wasting during cancer cachexia. Under cachectic conditions, tumor-derived cytokines and extracellular vesicles activate NF-κB, impair mitochondrial function, induce insulin resistance, and trigger proteolysis through upregulation of MuRF1 and Atrogin-1, collectively driving muscle wasting. In contrast, pharmacological activation of PPARγ promotes adiponectin secretion from adipocytes, which in turn activates AMPK and AKT signaling in muscle, thereby enhancing insulin sensitivity, stimulating anabolic pathways and mitochondrial biogenesis, and suppressing proteolysis and inflammation.

Beyond its direct anti-catabolic effects, PPARγ also enhances insulin sensitivity, which is essential for maintaining anabolic signaling and glucose utilization in skeletal muscle. Insulin resistance, commonly induced by tumor-derived factors, chronic inflammation, and endocrine dysfunction, is strongly associated with cancer-related weight loss and correlates positively with the severity of cachexia (27, 69, 70). By improving insulin responsiveness, PPARγ activation promotes glucose uptake and protein synthesis, thereby counteracting muscle wasting. However, not all findings are consistent, some investigations report that rosiglitazone fails to preserve muscle mass, strength, or proteolytic balance in cancer cachexia models (43, 71). Despite these discrepancies, additional data suggest that PPARγ contributes to mitochondrial health by stimulating biogenesis and restoring mitochondrial function, as demonstrated by the ability of PPARγ overexpression to reverse breast cancer-conditioned medium (BC-CM)-induced mitochondrial dysfunction (72) and by the effects of pioglitazone on mitochondrial biogenesis (73). Although these latter studies were not conducted in the context of cancer cachexia, they further support the concept that PPARγ activation may mitigate metabolic stress and preserve skeletal muscle integrity. In short, the current evidence positions PPARγ as a systemic and multifaceted regulator with therapeutic potential for combating muscle wasting in cancer cachexia (**Figure 3**).

### Anti-inflammatory role of PPARγ

Chronic systemic inflammation is widely recognized as a central driver of cancer cachexia, orchestrating muscle atrophy, adipose tissue remodeling, anorexia, insulin resistance, and metabolic dysregulation. Elevated levels of pro−inflammatory cytokines, particularly tumor necrosis factor−alpha (TNF−α), interleukin−6 (IL−6), and interleukin−1β (IL−1β), are consistently observed and are instrumental in activating catabolic pathways across peripheral tissues (1, 74). In this inflammatory milieu, PPARγ has emerged as a critical anti−inflammatory regulator with the potential to counteract cachexia−associated pathologies. PPARγ exerts its anti−inflammatory influence via transrepression of NF−κB and STAT3, two master regulators of inflammatory gene expression, and the modulation of their interaction appears necessary to suppress white adipose tissue inflammation in the context of cancer cachexia (75). Notably, activation of PPARγ has been shown to reduce phosphorylation of NF−κB and STAT3 both *in vitro* and *in vivo*, thereby disrupting the chronic inflammatory feedback loop that sustains cachexia progression (12, 76).

**Figure 4:**
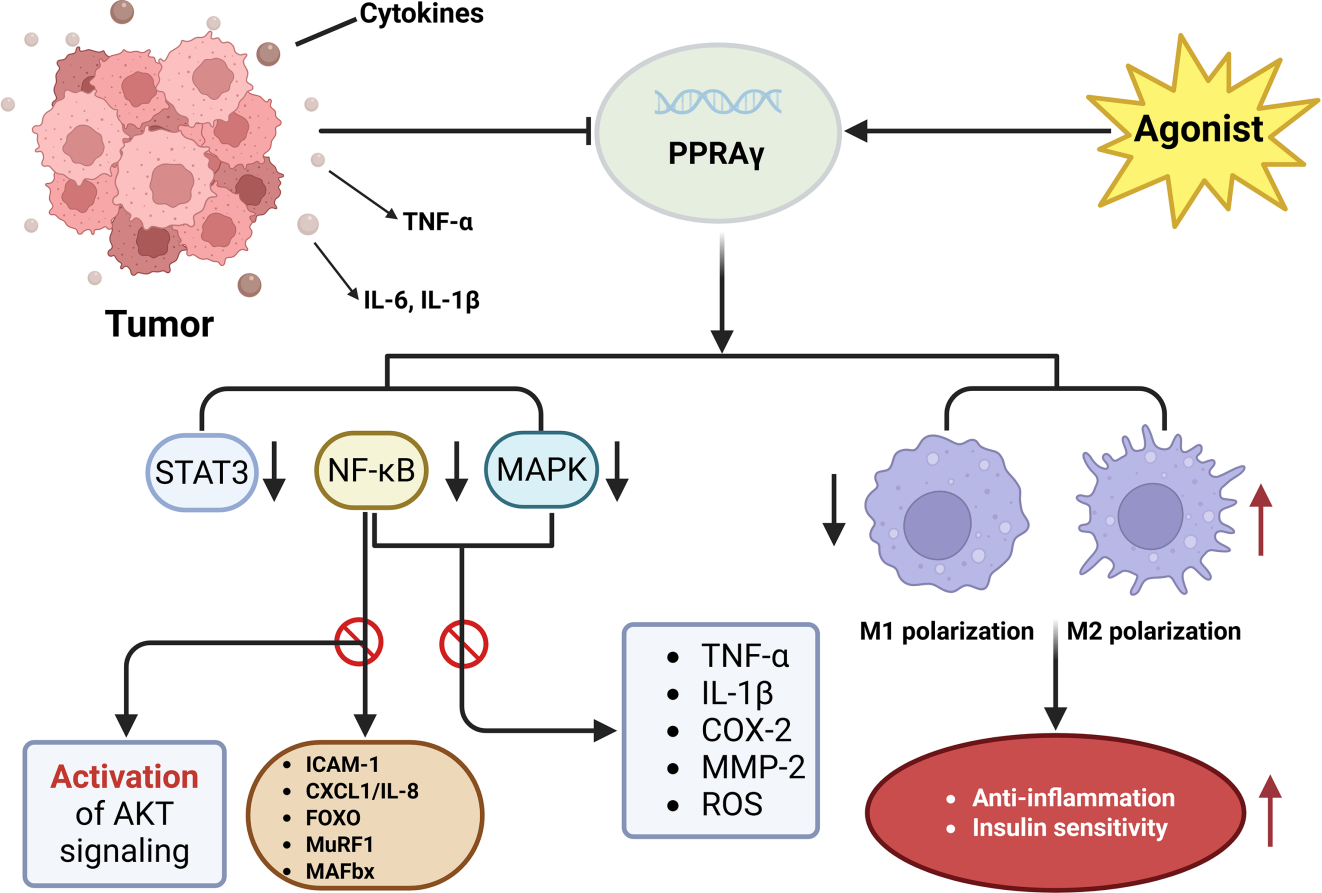
PPARγ counteracts systemic inflammation and tissue catabolism in cancer cachexia. Pharmacological activation of PPARγ suppresses NF-κB and STAT3 phosphorylation, thereby reducing proinflammatory cytokine and ROS production while simultaneously enhancing antioxidant defenses such as SOD and HO-1. In parallel, PPARγ downregulates FOXO-driven atrophy genes (e.g., MuRF1 and MAFbx) and promotes M2 macrophage polarization. Through these coordinated anti-inflammatory and immunomodulatory actions, PPARγ preserves metabolic homeostasis, attenuates systemic inflammation, and protects the structural integrity of both muscle and adipose tissues in cachexia.

In addition to its direct suppression of cytokine signaling, PPARγ exerts substantial immunomodulatory effects by regulating macrophage polarization and immune infiltration within adipose and skeletal muscle tissues. In adipose tissue, PPARγ is required for IL−4-induced M2 polarization, its deficiency shifts macrophage populations toward a pro−inflammatory M1 phenotype, thereby exacerbating local inflammation and impairing insulin sensitivity. Conversely, pro−inflammatory macrophages can suppress PPARγ activity in adipocytes through post−translational modifications such as S−nitrosylation, further reducing anti−inflammatory responses (77). Furthermore, macrophage−specific inactivation of PPARγ has been shown to amplify systemic inflammatory signaling, promote ectopic lipid accumulation, and worsen insulin resistance, underscoring the broader metabolic influence of macrophage−expressed PPARγ (78). In skeletal muscle, experimental overexpression or pharmacological activation of PPARγ in myotubes and animal models suppresses MAPK and NF−κB pathways, decreases the production of TNF−α, IL−1β, COX−2, MMP−2, and reactive oxygen species, while simultaneously increasing antioxidant enzymes including superoxide dismutase and heme−oxygenase−1 (79, 80). PPARγ activation also downregulates NF−κB-driven genes such as ICAM−1 and CXCL1/IL−8 (65), and in systemic inflammatory states, it protects AKT signaling, suppresses catabolic transcription factors such as FOXO, reduces the expression of muscle atrophy−related genes like MuRF1 and MAFbx, and preserves overall metabolic regulation (80), as shown in **Figure 4**. Here PPARγ functions as a protective ally in cancer cachexia by preserving skeletal muscle mass and mitigating inflammation through suppression of pro-inflammatory signaling cascades and modulation of immune cell dynamics. These multifaceted actions position PPARγ as a promising therapeutic target for alleviating the systemic inflammation and tissue catabolism that characterize cachexia.

### PPARγ and CNS homeostasis regulation

Cancer cachexia is a multifactorial syndrome characterized not only by profound peripheral tissue catabolism but also by significant disruptions in CNS homeostasis (5, 81). Among CNS regions, the hypothalamus plays a pivotal role in integrating peripheral inflammatory and metabolic cues and orchestrating adaptive or maladaptive responses that influence energy balance, feeding behavior, and metabolic regulation (82). In this context, PPARγ, although classically associated with adipose tissue and systemic metabolism, has emerged as a critical modulator within the CNS. PPARγ is expressed in various CNS cell types, including neurons, astrocytes, microglia, and oligodendrocytes, where it exerts anti-inflammatory and neuroprotective effects by suppressing microglial activation, reducing pro-inflammatory cytokine production, and supporting neuronal and astrocytic survival (30, 83, 84). Of translational relevance, central PPARγ activation, rather than adipose PPARγ, mediates TZD-induced weight gain through regulation of food intake and energy expenditure (30, 85, 86). Notably, the PPARγ agonist rosiglitazone has been shown to delay anorexia and attenuate adipose tissue loss in murine models of cancer cachexia, suggesting that central PPARγ signaling plays a protective role in mitigating anorexia and systemic wasting (43).

**Figure 5:**
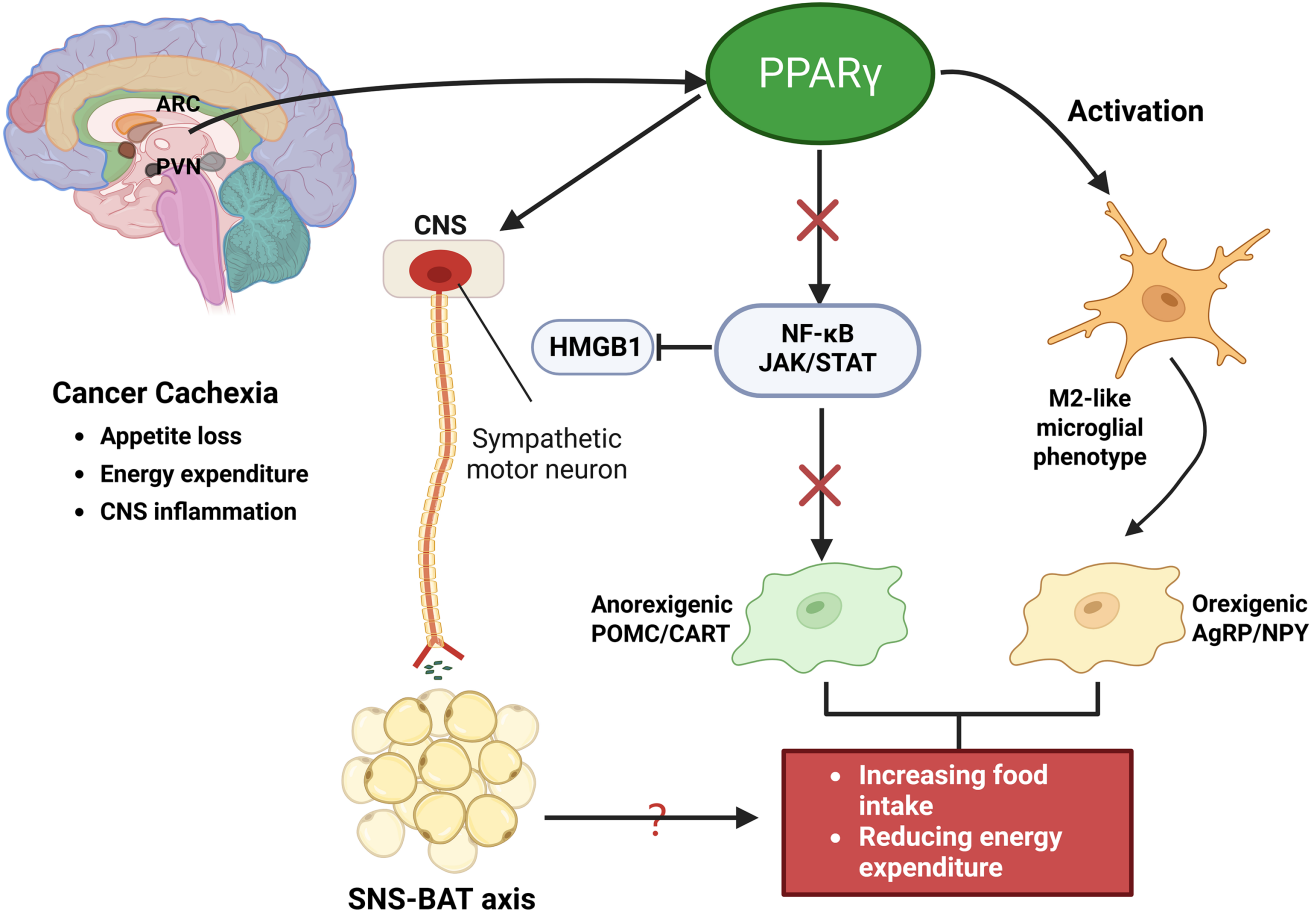
PPARγ regulation of CNS homeostasis in cancer cachexia. Activation of PPARγ suppresses NF-κB and JAK/STAT signaling, partly through inhibition of HMGB1, thereby reducing proinflammatory cytokine production and promoting an anti-inflammatory, M2-like microglial phenotype. Within hypothalamic centers such as the arcuate nucleus (ARC) and paraventricular nucleus (PVN), these effects preserve orexigenic AgRP/NPY neuronal activity while dampening anorexigenic POMC/CART signaling, thereby mitigating appetite loss and abnormal energy expenditure. In addition, PPARγ modulates sympathetic nervous system (SNS) output to BAT, regulating thermogenesis and contributing to systemic energy balance.

Direct evidence of hypothalamic PPARγ activity in cachexia is limited. However, key nuclei such as the ARC and PVN integrate hormonal and cytokine signals, including leptin, ghrelin, insulin, and IL-1β, to regulate orexigenic (AgRP/NPY) and anorexigenic (POMC/CART) neurons (87–89). In cachexia, elevated cytokines such as IL−1β and TNF−α inhibit NPY signaling and enhance anorexigenic drive, thereby contributing to reduced food intake and increased energy expenditure (90–92). These effects are accompanied by early microglial activation in the mediobasal hypothalamus (MBH), particularly the ARC and PVN, which exacerbates anorexia and muscle wasting (91, 93, 94). Interestingly, experimental depletion of microglia worsens cachexia, indicating that microglia may also exert compensatory or protective effects (94, 95). PPARγ activation counters these maladaptive responses by inhibiting NF−κB and JAK/STAT signaling, partly via suppression of HMGB1, and promoting an anti-inflammatory, M2-like microglial phenotype (96, 97). Through these mechanisms, PPARγ may preserve hypothalamic neuronal circuits essential for appetite and metabolic regulation. Moreover, hypothalamic PPARγ has been implicated in modulating sympathetic nervous system (SNS) output to brown adipose tissue (BAT), thereby influencing thermogenesis and systemic energy expenditure (46, 90, 98). Although further research is needed to clarify the precise role of hypothalamic PPARγ in SNS-BAT axis regulation during cachexia, current evidence supports its function as a central neuroimmune-metabolic integrator and a promising target for therapeutic intervention (**Figure 5**).

## Crosstalk with other metabolic regulators

PPARγ, a nuclear hormone receptor integral to lipid metabolism and adipogenesis, exerts multifaceted effects on energy homeostasis through its interactions with major metabolic regulators. In cancer cachexia, PPARγ may mediate a dynamic interplay with AMPK, mTOR, and PGC-1α, orchestrating the balance between anabolic and catabolic processes (**Table 1**).

**Table 1:** Crosstalk of PPARγ with metabolic regulators and nuclear receptors in cancer cachexia.

Crosstalk partner	Key functions	Interaction with PPARγ	Cachexia implications
AMPK	AMPK regulates FA oxidation, glucose uptake, glycolysis, autophagy, mitochondrial biogenesis, and insulin sensitivity (96).	PPARγ can activate AMPK in adipose tissue (96). AMPK enhances PPARγ deacetylation, thermogenic proteins (UCP-1) (98). Rosiglitazone increases AMPK and AKT in lung cancer models (Muscle Preservation) (13).	Muscle: Improves mitochondrial metabolism and preserves muscle mass. Adipose: Promotes lipolysis or fat loss (96, 97). Dual and tissue-specific role.
mTOR	mTOR regulates growth, protein synthesis, and autophagy suppression (99, 100).	In cachexia, chronic PPARγ activation and pioglitazone-induced AMPKα activation converge to suppress mTOR signaling (101, 102), impair protein synthesis, and promote autophagy through altered energy metabolism (↑ glycolysis, ↑ FA oxidation, ↓ ATP).	Positive: preserve muscle Negative: worsen metabolic dysregulation depending on tissue.
PGC-1α	PGC-1α: master regulator of mitochondrial biogenesis, OXPHOS, oxidative metabolism (103, 104).	PGC-1α coactivates PPARγ, and PPARγ agonists induce PGC-1α to enhance oxidative, lipid, and mitochondrial programs, while PGC-1α simultaneously suppresses FoxO-mediated catabolism (103, 105–107)	In muscle, reduced PGC-1α drives dysfunction and wasting, whereas activation of the PGC-1α axis restores mitochondrial homeostasis and protects against atrophy (108, 109).
PPARα / PPARδ	PPARα: FA β-oxidation, lipid metabolism (110). PPARδ: oxidative capacity, mitochondrial function (111).	LIF–STAT3 signaling suppresses PPARα to cause lipid imbalance, while fenofibrate restores lipid homeostasis and reduces cachexia, and PPARδ activation enhances oxidative metabolism and muscle endurance (112–114).	Isoform-specific modulation critical. PPARα loss: worsens lipid depletion. PPARδ activation: combats muscle wasting.
LXR	LXRs: regulate cholesterol and lipid homeostasis, function as RXR heterodimers (115).	LXR signaling suppresses macrophage proliferation (116), promotes lipid accumulation in muscle, induces fatty acid synthesis and lipotoxicity risk (118), yet LXR agonists reduce muscle wasting in preclinical models (119).	Positive: Anti-inflammatory, anti-wasting. Negative: Ectopic lipid deposition. Outcomes context-dependent.
RAR (via RXR)	RARs and PPARγ both heterodimerize with RXR.	RXR competes for binding partners, but RXRα agonists (e.g., SR11237) (120) and oxime rexinoids promote RXR:PPARγ dimerization to drive adipogenesis (121–123) , while RXR:PPARγ signaling regulates glucose and fatty acid metabolism in muscle (124, 125).	Adipose: RXR:PPARγ promotes adipogenesis. Muscle: RXR:PPARγ supports metabolic function. Tissue dependent.
GR	GRs regulate proteolysis, adipose catabolism; drivers of inflammation-induced atrophy.	GR signaling promotes tumor-induced atrophy, as GR knockout confers protection (126); pioglitazone exerts partial anti-inflammatory effects via GR (127), while the GR antagonist mifepristone (also a partial PPARγ agonist) induces adipogenesis (128).	Muscle: GR indispensable for atrophy. Adipose: GR–PPARγ crosstalk affects remodeling; ligand-specific.

### PPARγ and AMPK

AMPK serves as a central regulator of cellular energy homeostasis, orchestrating key metabolic processes such as fatty acid oxidation, glucose uptake, glycolysis, autophagy, mitochondrial biogenesis and degradation, as well as insulin sensitivity to maintain intracellular ATP levels (99). In the context of cancer cachexia, AMPK is frequently activated in both skeletal muscle and adipose tissue, where it contributes to enhanced fatty acid oxidation and mitochondrial biogenesis, while simultaneously inhibiting protein synthesis, thereby exacerbating muscle wasting and lipid depletion (99, 100). The interplay between AMPK and PPARγ is complex and context-dependent, exhibiting both synergistic and antagonistic dynamics. On one hand, activation of PPARγ has been shown to stimulate AMPK signaling in adipose tissue, indicating a bidirectional regulatory loop. Conversely, AMPK can enhance the deacetylation of PPARγ, thereby promoting adipose tissue remodeling and upregulating thermogenic proteins such as UCP-1 (101). In skeletal muscle, this axis may exert protective effects by improving mitochondrial metabolism and preserving muscle mass, as evidenced by rosiglitazone-induced increases in both AMPK and AKT activity in lung cancer models (11). However, in adipose tissue, the same axis may facilitate energy mobilization and lipolysis, potentially worsening fat loss. Therefore, the AMPK–PPARγ signaling axis may play dual and tissue-specific roles in cancer cachexia, acting as a double-edged sword that may be beneficial in muscle preservation while simultaneously contributing to adipose tissue wasting.

### PPARγ and mTOR

The mTOR is a central regulator of cellular growth, nutrient sensing, and protein synthesis, critically involved in maintaining skeletal muscle mass and preventing atrophy. In cancer cachexia, mTOR signaling is frequently downregulated, leading to impaired muscle protein synthesis and enhanced autophagic activity, both of which contribute significantly to progressive muscle wasting (102, 103). Notably, the interplay between PPARγ and mTOR signaling occurs via complex metabolic reprogramming pathways. For instance, treatment with the PPARγ agonist pioglitazone has been shown to reduce tumor growth in a metastatic PC3 prostate cancer xenograft model, accompanied by increased phosphorylation of AMPKα and decreased phosphorylation of mTOR. This metabolic shift was further characterized by pioglitazone-induced enhancement of glycolysis in primary prostate cancer cells and increased fatty acid oxidation in metastatic cells, ultimately resulting in reduced mitochondrial ATP production (104). Moreover, chronic PPARγ activation in adipose tissue may promote excessive lipid accumulation and metabolic dysregulation, potentially suppressing mTOR signaling indirectly through lipotoxic mechanisms (105). Together, these findings highlight the multifaceted relationship between PPARγ activity and mTOR signaling, suggesting that modulation of this axis could have both therapeutic potential and context-dependent metabolic consequences in cancer cachexia.

### PPARγ and PGC-1α

PGC-1α is a master regulator of mitochondrial biogenesis, oxidative phosphorylation, and energy metabolism, particularly in metabolically active tissues such as skeletal muscle (106, 107). In muscle physiology, PGC-1α confers protection against atrophy by enhancing mitochondrial function, promoting oxidative fiber-type switching, and suppressing catabolic signaling pathways such as the FoxO family of transcription factors (108). Notably, PGC-1α functions as a transcriptional coactivator for multiple NRs, including PPARγ, thereby establishing a synergistic axis critical for maintaining mitochondrial integrity and metabolic flexibility (106, 109). PPARγ agonists have been shown to induce PGC-1α expression, which in turn potentiates PPARγ-driven transcriptional programs involved in adipocyte differentiation, lipid metabolism, and oxidative gene expression (109, 110). This reciprocal regulatory relationship underscores the therapeutic potential of targeting the PPARγ–PGC-1α axis in metabolic disorders. In cancer cachexia, diminished PGC-1α expression in skeletal muscle has been consistently associated with mitochondrial dysfunction, impaired oxidative capacity, and exacerbated muscle wasting (111, 112). Thus, pharmacological activation of this signaling axis may offer a promising strategy to restore mitochondrial homeostasis, preserve muscle mass, and improve functional outcomes in cachectic patients.

## Crosstalk with other NRs

PPARγ does not function in isolation but rather interacts dynamically with other NRs to orchestrate complex metabolic programs. In cancer cachexia, these interactions may significantly modulate the disease trajectory (**Table 1**). Understanding the crosstalk between PPARγ and other NRs is essential for deciphering its dualistic role in cachexia.

### PPARγ and PPARα/δ

Among the three PPAR isoforms, PPARγ, PPARα, and PPARδ exhibit overlapping yet distinct functions in metabolic regulation. PPARγ is primarily responsible for promoting adipogenesis, lipid uptake, and storage, whereas PPARα and PPARδ serve as key regulators of fatty acid β-oxidation, mitochondrial biogenesis, and oxidative metabolism (113, 114). Recent evidence indicates that leukemia inhibitory factor (LIF)-induced activation of STAT3 suppresses hepatic PPARα expression, resulting in downregulation of its target genes involved in lipid metabolism and contributing to reduced hepatic lipogenesis and systemic lipid imbalance (115). Pharmacological activation of PPARα with the agonist fenofibrate restores hepatic lipid homeostasis and mitigates LIF-induced cachexia, underscoring the importance of isoform-specific regulation in maintaining metabolic integrity (115). While such metabolic reprogramming may initially represent an adaptive response to energy stress, sustained suppression of oxidative and lipogenic pathways ultimately accelerates the loss of adipose and muscle mass. In contrast, activation of PPARδ enhances mitochondrial function and oxidative capacity in skeletal muscle, improving endurance and potentially counteracting muscle atrophy (116, 117). Therefore, therapeutic strategies that restore PPARγ activity or selectively modulate PPARα and PPARδ signaling may offer a promising avenue to reestablish systemic metabolic balance and attenuate tissue wasting in cachectic conditions.

### PPARγ and liver X receptors

LXRs, key NRs governing cholesterol homeostasis and lipid metabolism, function as heterodimers with retinoid X receptors (RXRs), a structural and mechanistic feature they share with PPARγ (118). Upon activation, LXRs suppress macrophage proliferation through transcriptional regulation of cyclins and cyclin-dependent kinases (119), and concurrently promote lipid accumulation in human skeletal muscle cells (120), activities that both overlap with and sometimes counteract the metabolic roles of PPARγ. Notably, the LXR-mediated induction of stearoyl-CoA desaturase 1 (SCD1) and *de novo* fatty acid synthesis has emerged as a significant barrier to the therapeutic exploitation of these receptors, particularly in metabolic disorders (121). In cancer cachexia, LXR activation presents a double-edged sword: while it may attenuate systemic inflammation, it can also exacerbate ectopic lipid deposition, thereby complicating the overall metabolic profile. Nevertheless, evidence from certain preclinical models suggests that LXR agonists can ameliorate muscle wasting (122), highlighting the therapeutic potential of co-targeting LXR and PPARγ pathways to combat tissue catabolism while maintaining lipid homeostasis.

### PPARγ and retinoic acid receptors

PPARγ and RARs competitively bind to RXR, which serves as a common heterodimerization partner, thus creating a regulatory nexus between distinct nuclear receptor pathways (123). The RXRα agonist SR11237, although inactive on PPARγ itself, promotes the formation of the PPARγ:RXRα heterodimer by destabilizing RXRα homodimers and redirecting RXRα oligomerization preference toward PPARγ (123). This ligand-induced shift indirectly enhances coactivator recruitment to PPARγ, thereby modulating its transcriptional activity without directly activating or inhibiting the receptor. In adipocytes, certain oxime rexinoids and transcription factors act as agonists of the RXR: PPARγ heterodimer and are potent inducers of 3T3-L1 preadipocyte differentiation into mature adipocytes (124–126), implicating this axis in adipose tissue remodeling and potentially contributing to lipid depletion observed in cancer cachexia. Conversely, PPARγ and RXR agonists also regulate glucose and free fatty acid metabolism in human skeletal muscle (127, 128), highlighting a tissue-specific functional divergence of RXR: PPARγ signaling. Together, these findings underscore the context-dependent outcomes of RXR modulation, wherein the competition between PPARγ and RARs for RXR binding and subsequent heterodimerization may either exacerbate adipose loss or preserve metabolic function during cachectic progression, depending on cellular context and ligand availability.

### PPARγ and glucocorticoid receptors

Glucocorticoids are potent regulators of both muscle proteolysis and adipose tissue catabolism, processes that are markedly amplified in cancer cachexia. As key mediators of inflammation-induced atrophy *in vivo*, glucocorticoids contribute significantly to the pathogenesis of cancer-associated muscle wasting. In support of this, muscle-specific GR knockout mice exhibit protection against tumor-induced muscle atrophy following Lewis lung carcinoma (LLC) inoculation, underscoring the indispensable role of GR signaling in cachectic muscle degradation (129). However, GR activity does not directly participate in PPARγ/RXR heterodimer signaling. Interestingly, the PPARγ agonist pioglitazone exerts only partial anti-inflammatory effects via GR activation, suggesting limited cross-activation between these nuclear receptor pathways (130). Conversely, mifepristone, a GR antagonist with partial PPARγ agonist activity, induces adipocyte differentiation both *in vitro* (3T3-L1 cells) and *in vivo*, with PPARγ playing a critical role in mediating these effects (131). Together, these findings reveal that while GR signaling is indispensable for the muscle catabolism observed in cancer cachexia, its interaction with PPARγ pathways in adipose tissue remodeling is more nuanced and may involve ligand-specific or tissue-specific regulatory mechanisms.

## PPARγ in cancer progression

**Figure 6:**
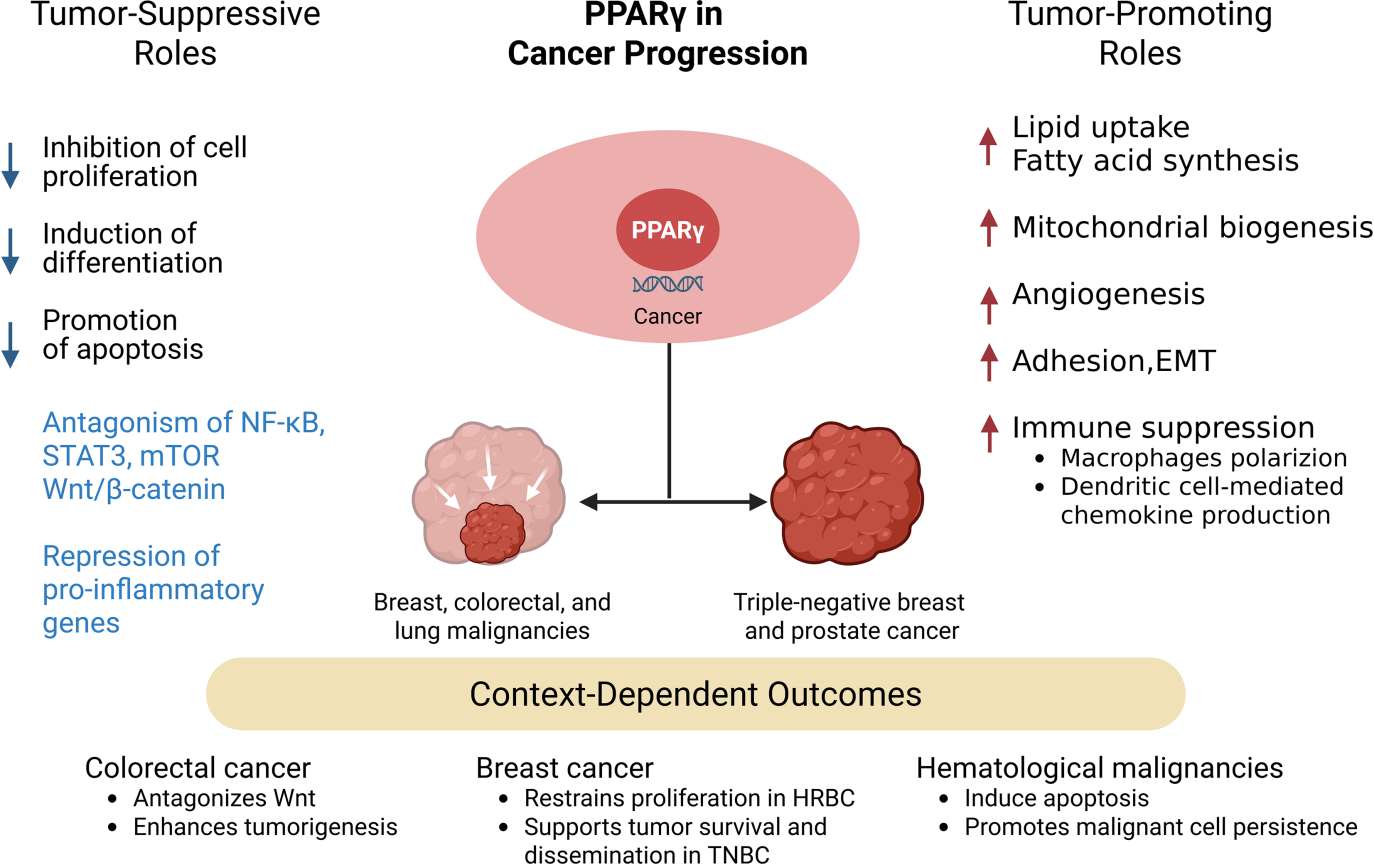
Dual and context-dependent roles of PPARγ in cancer progression. PPARγ activation exerts both tumor-suppressive and tumor-promoting effects that are highly dependent on tumor type and microenvironmental context. On the suppressive side, PPARγ inhibits cell proliferation, promotes differentiation and apoptosis, antagonizes oncogenic pathways such as NF-κB, STAT3, mTOR, and Wnt/β-catenin, and represses proinflammatory gene expression. Conversely, PPARγ may facilitate tumor progression by enhancing lipid metabolism and mitochondrial biogenesis, stimulating angiogenesis, promoting adhesion and epithelial–mesenchymal transition (EMT), and shaping the immune microenvironment toward immunosuppression. These dual outcomes reflect the influence of tumor subtype and microenvironmental cues in determining the net impact of PPARγ activity.

In cancer, PPARγ has a dual role, sometimes acting as a tumor suppressor, other times as a tumor promoter, depending on the context (**Figure 6**) (132).

### Tumor-suppressive roles

PPARγ has emerged as a multifaceted regulator of tumor biology, exhibiting both tumor-suppressive and tumor-promoting activities. On the one hand, activation of PPARγ by synthetic agonists such as TZDs or natural ligands like resveratrol and curcumin can inhibit cell proliferation, induce differentiation, and promote apoptosis across various cancer types including breast, colorectal, and lung malignancies (132, 133). Mechanistically, PPARγ antagonizes oncogenic pathways such as NF-κB (134), STAT3 (135), mTOR (136)and Wnt/β-catenin (132, 137), while repressing pro-inflammatory gene expression through SUMOylation-dependent trans-repression (31, 134). These effects not only impair tumor cell survival but also reduce metastatic signaling, as illustrated by the downregulation of chemokine receptor CXCR4 in breast cancer (138). Clinically, higher PPARγ expression has been correlated with improved prognosis in certain tumor contexts, and epigenetic derepression of PPARG has been associated with enhanced therapeutic responsiveness (132), highlighting its potential as a favorable prognostic biomarker and therapeutic target.

### Tumor-promoting roles

However, accumulating evidence indicates that PPARγ can also facilitate tumor progression in specific contexts, particularly in metabolically adaptive or advanced-stage cancers. Through regulation of lipid uptake, fatty acid synthesis, and mitochondrial biogenesis, PPARγ provides metabolic flexibility that supports rapid tumor growth and survival under stress (139). PPARγ has been shown to support prostate cancer growth through its roles in fatty acid synthesis, mitochondrial biogenesis, and co-operating with androgen receptor signaling (139), while in triple-negative breast cancer its activity strengthens lipid metabolic networks that underlie invasive behavior (140). Moreover, PPARγ activation can enhance angiogenesis through VEGF-A induction, increase cellular adhesion via carcinoembryonic antigen expression, and foster epithelial-mesenchymal transition (EMT) through vimentin upregulation (141). Beyond tumor-intrinsic effects, PPARγ signaling may reshape the tumor microenvironment by polarizing macrophages toward immunosuppressive phenotypes and attenuating dendritic cell-mediated chemokine production (30, 142), thereby weakening anti-tumor immunity. In this context it may also function as a foe by supporting tumor metabolism. These findings underscore the dual nature of PPARγ activity, raising concerns about long-term PPARγ agonist use and suggesting that its tumor-promoting functions may override its suppressive potential under certain conditions.

### Paradoxical roles

The contrasting roles of PPARγ in cancer progression reflect its strong context dependency, with outcomes dictated by tumor type, molecular alterations, and microenvironmental cues. For example, in colorectal cancer, PPARγ activation can antagonize Wnt-driven oncogenesis, yet in APC-mutant settings it may paradoxically enhance tumorigenesis (132). In hormone receptor-positive breast cancers, PPARγ activity generally restrains proliferation, whereas in aggressive triple-negative subtypes it supports metabolic reprogramming that fuels tumor survival and dissemination (132, 138). Similarly, in hematological malignancies, PPARγ activation may induce apoptosis through inhibition of Akt/mTOR signaling (143), but in other settings it promotes malignant cell persistence depending on differentiation status. Thus, PPARγ represents both a potential therapeutic ally and a liability, demanding a nuanced and tumor-specific approach. Ultimately, the integration of molecular subtype, stage, and metabolic state will be critical in determining whether targeting PPARγ, through activation or inhibition, can be leveraged for therapeutic benefit in oncology.

## Therapeutic potential of PPARγ agonists in cancer cachexia

PPARγ agonists, particularly the TZD class, have long been recognized for their insulin-sensitizing and anti-inflammatory properties in metabolic diseases. Their potential to modulate skeletal muscle wasting, adipose tissue browning, systemic inflammation, and energy metabolism has recently attracted attention in the context of cancer cachexia. Given the multifactorial nature of cachexia, encompassing muscle atrophy, lipolysis, anorexia, and chronic inflammation, PPARγ agonists offer a mechanistically diverse therapeutic strategy that targets several hallmarks of this syndrome (**Table 2**). While TZDs delay wasting in preclinical models, clinical application is limited by side effects and tumor concerns.

**Table 2:** Therapeutic opportunities of PPARγ Agonists in cancer cachexia.

Agonist (class)	Muscle preservation and anti-catabolic effects	Modulation of adipose tissue remodeling	Anti-inflammatory functions	Appetite stimulation	Key cachexia evidence (model)	References
Rosiglitazone (TZD, full agonist)	Preserves skeletal muscle mass; Activates AMPK/AKT signaling; Lowers markers of cachexia.	Preserves adipose tissue; Restores circulating adiponectin in lung cancer cachexia.	Attenuates cachexia-related inflammation.	Delays anorexia and body weight loss.	Two preclinical studies (C26 mice, Kras/Lkb1 mouse model).	(13, 29)
Pioglitazone (TZD, full agonist)	Prevents body weight loss and improves survival in tumor-bearing rats	Improves adipose mass and insulin sensitivity in Walker-256 cachexia; systems-level remodeling noted in tumor-bearing mice.	Insulin-sensitizing; metabolic anti-inflammatory effects tied to PPARγ activation.	Increases food intake (rodents) and energy intake (humans), consistent with appetite/metabolic benefit.	Preclinical (Walker-256 rats); Phase II trial ongoing in lung-cancer cachexia (NCT05919147).	(141–145)
Telmisartan (ARB, partial PPARγ agonist/ SPPARM)	Reduces muscle atrophy in chemo-treated cachexia models when combined with cisplatin/5-FU.	Mitigates adipose loss and dyslipidemia in rat models (combination therapy).	Lowers IL-6/oxidative stress; AMPK–mTOR effects reported (anti-proliferative; PPARγ-modulation).	Metabolic improvements (glucose/lipids) reported alongside anti-wasting effects in rats.	Preclinical (oral/oral+chemo; oral/oral cancer cachexia in rats).	(146, 147)
Alpinetin (natural flavonoid, PPARγ-dependent)	Alleviates muscle wasting via PPARγ activation; suppresses MuRF1/Atrogin-1; effect blocked by PPARγ antagonist GW9662.	May limit adipose catabolism indirectly via anti-inflammatory signaling.	Inhibits NF-κB and STAT3 phosphorylation in cachexia models (PPARγ-dependent).	Not primarily orexigenic; metabolic benefit via PPARγ signaling.	Preclinical (LLC/C26 models).	(14)
15-deoxy-Δ12,14-PGJ2 (15d-PGJ2) (endogenous cyclopentenone prostaglandin, PPARγ agonist)	Indirect support for myogenesis and muscle homeostasis; emerging data that 15d-PGJ2 from senescent cells modulates muscle differentiation after chemotherapy.	Classical PPARγ ligand; can influence adipocyte programs (context-dependent).	Potent anti-inflammatory actions (NF-κB/JAK-STAT/Nrf2 pathways), sometimes PPARγ-independent.	No clear appetite data; potential metabolic effects via PPARγ/Nrf2.	Mechanistic and preclinical; limited direct cachexia outcome data to date.	(148, 149)
Omega-3 (EPA/DHA) (nutritional PPARγ-activating ligands)	Mixed: some preclinical anti-wasting signals; human trials largely mixed for weight/lean mass.	May modulate WAT inflammation/fibrosis and promote favorable adipocyte signaling via PPARγ.	Broad anti-inflammatory activity (↓ NF-κB), partly via PPARγ.	QoL/survival benefits reported in meta-analysis despite little effect on weight.	Clinical evidence heterogeneous (pancreatic and mixed-cancer cohorts).	(150–152)

### Rosiglitazone

Among TZDs, rosiglitazone has been the most extensively investigated in experimental models of cancer cachexia. In male CD2F1 mice bearing colon-26 adenocarcinoma, rosiglitazone administration increased serum APN levels, improved insulin sensitivity, and promoted body weight gain. These effects were accompanied by reduced expression of the muscle-specific E3 ligases Atrogin-1 and MuRF-1, together with attenuation of early cachexia markers (27). More recent studies in lung cancer cachexia models have confirmed and extended these findings. Rosiglitazone treatment delayed weight loss and preserved both skeletal muscle and adipose tissue mass compared with vehicle-treated controls. Muscle preservation was associated with enhanced AMPK and AKT signaling. In line with these results, direct activation of APN receptors in muscle cells also stimulated AMPK activity, increased anabolic signaling, and promoted protein synthesis, thereby supporting rosiglitazone’s dual action on metabolic regulation and structural maintenance (11). Collectively, these findings indicate that rosiglitazone exerts multifaceted anti-cachectic effects. By improving insulin sensitivity, enhancing anti-catabolic signaling, and reducing systemic inflammation, rosiglitazone contributes to both metabolic stability and muscle preservation, highlighting its potential therapeutic relevance in cancer cachexia.

### Pioglitazone

Pioglitazone, another TZD, has demonstrated potent protective effects in rat models of tumor-induced cachexia. In Walker-256 tumor-bearing rats, pioglitazone administration prevented body weight loss, preserved muscle, and prolonged survival. Mechanistically, these effects were associated with improvements in insulin sensitivity and adipose tissue remodeling, mitigating systemic catabolism (144). Notably, pioglitazone also stimulated food intake in rodents and increased energy intake in human subjects, suggesting both metabolic and orexigenic benefits (144–148). Building on these findings, a Phase II clinical trial (NCT05919147) is currently investigating pioglitazone in lung-cancer patients with cachexia, positioning it as one of the few PPARγ agonists advancing into clinical evaluation.

### Telmisartan

Telmisartan, an angiotensin II receptor blocker with partial PPARγ agonist activity, represents a pharmacologically distinct approach to cachexia management. Preclinical studies combining telmisartan with cisplatin or 5-fluorouracil showed marked attenuation of muscle atrophy in rat models of oral and gastric cancer. Beyond skeletal muscle preservation, telmisartan ameliorated adipose tissue loss, improved lipid handling, and reduced circulating IL-6 and oxidative stress markers. Mechanistically, these outcomes reflect activation of AMPK/mTOR pathways and immunomodulatory functions attributed to partial PPARγ engagement (149, 150). By simultaneously targeting tumor progression, systemic inflammation, and tissue catabolism, telmisartan exemplifies how selective PPARγ modulation could be therapeutically exploited in cachexia.

### Alpinetin

Alpinetin, a naturally occurring flavonoid, has emerged as a promising PPARγ-dependent modulator of cancer cachexia. In Lewis lung carcinoma and C26 tumor models, alpinetin alleviated skeletal muscle wasting through direct activation of PPARγ, as evidenced by reduced MuRF1 and Atrogin-1 expression. The specificity of this effect was confirmed using the PPARγ antagonist GW9662, which abolished alpinetin’s protective influence. In addition to muscle preservation, alpinetin suppressed phosphorylation of NF-κB and STAT3, key mediators of cachexia-associated inflammation (12). These results position alpinetin as a potential nutraceutical or adjuvant therapeutic agent capable of targeting both metabolic and inflammatory aspects of the syndrome.

### 15d-PGJ2

15d-PGJ2, an endogenous cyclopentenone prostaglandin and natural PPARγ agonist, has been implicated in muscle differentiation and repair following chemotherapy-induced tissue damage. Although direct cachexia outcome data remain limited, preclinical studies suggest that 15d-PGJ2 may contribute to myogenic homeostasis while exerting broad anti-inflammatory effects via NF-κB, JAK-STAT, and Nrf2 signaling pathways. Importantly, some of these activities are PPARγ-independent, underscoring its pleiotropic pharmacology. The compound also influences adipocyte differentiation and lipid metabolism, though the relevance of these pathways to cancer cachexia requires further elucidation. As such, 15d-PGJ2 remains mechanistically intriguing but translationally underexplored in the context of cachexia (151, 152).

### Omega-3 polyunsaturated fatty acids (EPA/DHA)

Dietary omega-3 fatty acids, particularly eicosapentaenoic acid (EPA) and docosahexaenoic acid (DHA), can act as nutritional ligands for PPARγ and have been investigated in numerous cachexia studies. Preclinical data suggest benefits on muscle preservation and adipose tissue inflammation; however, randomized clinical trials have yielded mixed outcomes with respect to weight or lean body mass. Meta-analyses indicate that while omega-3 supplementation often fails to reverse weight loss, it may improve quality of life and survival, possibly through anti-inflammatory effects mediated in part by PPARγ (153–155). Thus, omega-3s may serve best as supportive agents in multimodal cachexia management rather than as stand-alone therapies.

## Future directions and challenges

Despite substantial progress, the role of PPARγ in cancer cachexia remains incompletely defined due to its tissue-specific and context-dependent actions. In skeletal muscle, cachexia is associated with altered expression of PPAR isoforms, including PPARγ, along with metabolic regulators such as PGC-1α and PDK4. In the Yoshida AH-130 hepatoma rat model, these changes were reversed by β_2_-agonist treatment, suggesting that PPARγ contributes to muscle metabolic reprogramming and atrophy (117). In adipose tissue, PPARγ is a master regulator of lipid storage and adipogenesis, yet cancer cachexia is characterized by enhanced lipolysis, impaired lipogenesis, and white adipose tissue catabolism, leading to systemic energy imbalance (156). Moreover, in the tumor microenvironment and immune compartments, PPARγ exerts highly variable effects, modulating inflammation, differentiation, and metabolism in ways that may either support or suppress cachexia progression, depending on cancer type and inflammatory milieu (132).

From a therapeutic standpoint, PPARγ agonists such as TZDs have demonstrated modest benefits in preclinical models, including delayed body weight loss and preservation of muscle and fat mass via activation of AMPK/AKT pathways (11). However, their clinical applicability is hampered by adverse effects, including fluid retention, cardiovascular risk, and potential tumor-promoting actions (157). These safety issues highlight the limitations of full PPARγ activation in cachectic cancer patients. SPPARγMs represent a promising alternative, offering the potential to dissociate metabolic benefits from off-target toxicities, but their role in cancer cachexia remains largely unexplored and represents a critical research gap. Recent pharmacological advances emphasize that the biological outcomes of PPARγ activation are not only ligand-dependent but also determined by differences in pharmacokinetic distribution, including lipophilicity, tissue affinity, and clearance rates, which collectively shape the exposure of PPARγ agonists in different organs (158–161). Thus, rational optimization of ligand structure and delivery strategies could achieve tissue-preferential activation and minimize systemic toxicity (162, 163).

Emerging approaches in tissue-specific drug delivery provide additional opportunities to refine PPARγ-targeted therapy for cachexia. Nanoparticle- or macromolecule-based delivery systems, for instance, can preferentially accumulate in skeletal muscle (164) or adipose compartments (165, 166), allowing localized PPARγ activation while sparing tissues such as the liver or kidney, where its overactivation may induce metabolic stress or fluid imbalance (167–169). Incorporating such strategies could enhance the therapeutic window of PPARγ agonists, maintaining their anti-catabolic and anti-inflammatory effects in muscle and fat while avoiding counterproductive actions elsewhere (144, 146). Future preclinical studies integrating pharmacokinetic profiling with nanocarrier-based or ligand-engineering strategies will be essential to optimize this selective modulation for clinical translation.

An additional challenge lies in mapping the extensive signaling cross-talk between PPARγ and other cachexia-relevant pathways. In muscle, PPARγ and its coactivator PGC-1α interact with regulators of mitochondrial metabolism, suggesting overlap with AKT and AMPK signaling (117). Yet, how PPARγ modulation influences inflammatory networks governed by STAT3, NF-κB, and FOXO1 remains poorly understood, despite these pathways being central to muscle wasting and adipose dysfunction (51). Addressing this complexity will require systems biology approaches that integrate transcriptomic, metabolomic, and phospho-proteomic data to construct multi-layered interaction maps, enabling rational design of combination therapies that target multiple nodes of the cachectic signaling network.

Finally, translating PPARγ-based strategies to the clinic demands rigorous validation in human settings. Most mechanistic insights derive from murine models, which fail to capture the heterogeneity of human cachexia. Clinical trials will need to account for tumor type, disease stage, metabolic phenotype, and comorbidities, while incorporating biomarkers such as circulating adipokines, lipid mediators, and gene expression signatures for patient stratification and therapeutic monitoring (1). Incorporating tissue-targeted delivery systems and pharmacodynamic monitoring may further improve clinical predictability, ensuring that beneficial PPARγ activation occurs in the intended tissues. Emerging tools, such as multimodal biomarker platforms that combine molecular data with imaging and clinical notes, offer promising avenues for early detection and precision targeting (170). Ultimately, advancing the field will require a multidisciplinary effort spanning molecular biology, pharmacology, oncology, and clinical nutrition to fully harness the therapeutic potential of PPARγ in cancer cachexia.

## Conclusion

**Figure 7:**
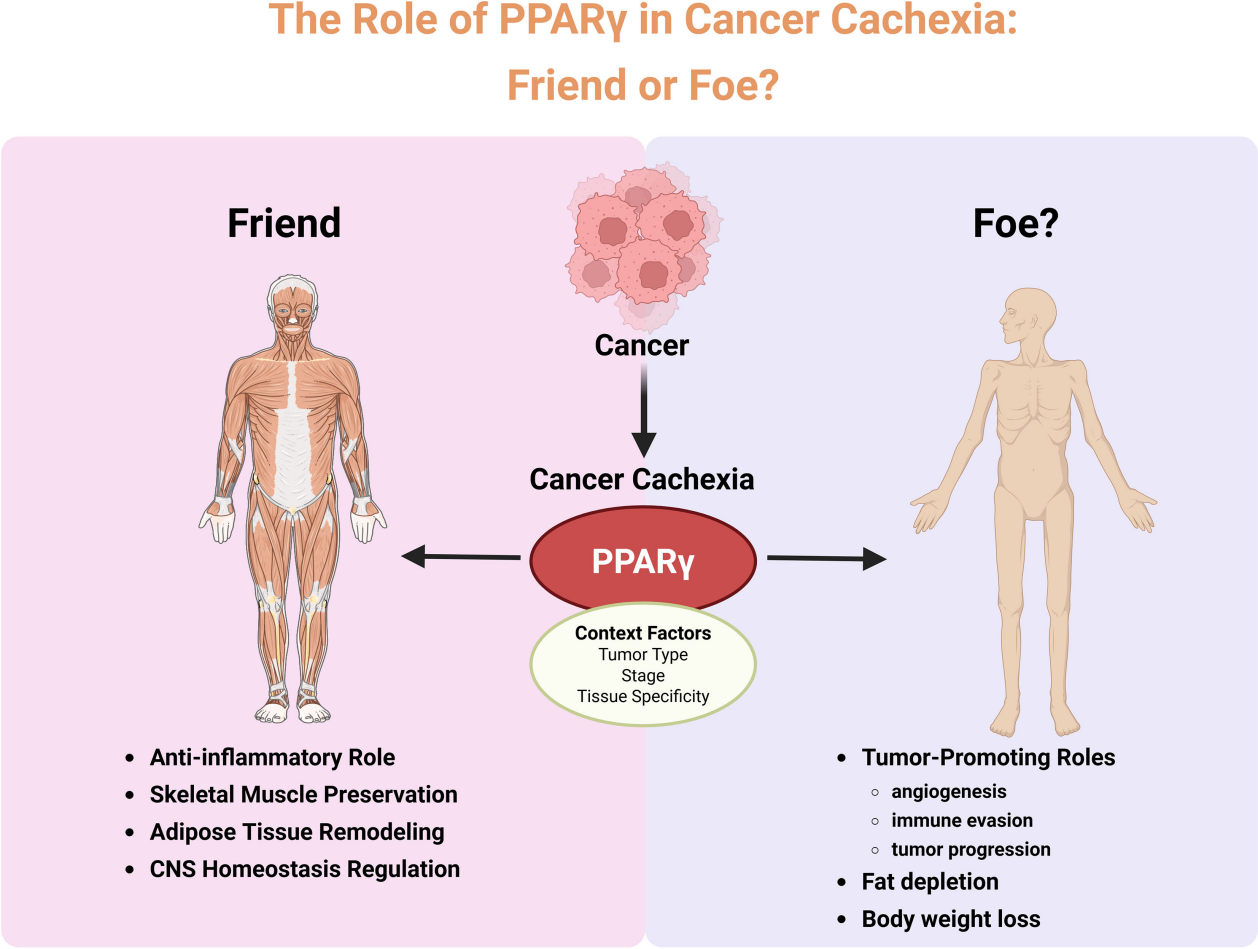
The dual role of PPARγ in cancer cachexia. This schematic illustrates the context-dependent effects of PPARγ in cancer cachexia. In the setting of cancer, PPARγ activity can exert protective (“friend”) functions-including anti-inflammatory effects, preservation of skeletal muscle mass, remodeling of adipose tissue, and regulation of central nervous system (CNS) homeostasis-thereby mitigating cachexia progression. Conversely, under certain tumor-specific contexts, PPARγ may display deleterious (“foe”) roles, contributing to tumor-promoting processes such as angiogenesis, immune evasion, and tumor progression, ultimately leading to fat depletion and body weight loss. The net effect of PPARγ on cachexia outcomes is influenced by contextual factors such as tumor type, stage, and tissue specificity.

Cancer cachexia remains a major unmet challenge in oncology, marked by progressive muscle wasting, adipose depletion, and systemic inflammation. PPARγ occupies a central but paradoxical position in this syndrome. On one hand, its activation suppresses lipolysis, limits white adipose browning, enhances insulin sensitivity, preserves skeletal muscle integrity, and attenuates inflammatory cascades. On the other, PPARγ can be co-opted by tumors to promote metabolic adaptation, angiogenesis, and immune evasion, raising concerns about its therapeutic exploitation. These dual roles underscore the importance of context. The outcome of PPARγ modulation depends on cancer type, disease stage, tissue specificity, and the surrounding inflammatory and metabolic environment (**Figure 7**). While classical PPARγ agonists such as TZDs show preclinical promise, their clinical translation is hindered by safety concerns and potential tumor-promoting activity. Emerging approaches, including SPPARγMs, tissue-specific targeting, and rational combination therapies, offer a path forward to maximize anti-cachectic benefits while minimizing oncogenic risks. Future research should prioritize integrative approaches that combine molecular biology, multi-omics profiling, and precision medicine. By disentangling the tissue- and context-specific actions of PPARγ, it may be possible to transform this controversial receptor from a double-edged sword into a clinically actionable target for the management of cancer cachexia.
